# 0Phobia – towards a virtual cure for acrophobia: study protocol for a randomized controlled trial

**DOI:** 10.1186/s13063-018-2704-6

**Published:** 2018-08-09

**Authors:** T. Donker, S. Van Esveld, N. Fischer, A. Van Straten

**Affiliations:** 1 Department of Clinical, Neuro and Developmental Psychology, Faculty of Movement- and Behavioral Sciences, Section Clinical Psychology, Vrije Universiteit Amsterdam, Amsterdam, The Netherlands; 2 Amsterdam Public Health Research Institute, Vrije Universiteit Amsterdam, Van der Boechorststraat 7, 1081 BT Amsterdam, The Netherlands; 3 Faculty Governance and Global Affairs, Leiden University, Leiden, The Netherlands

**Keywords:** Acrophobia, Specific phobia, Virtual reality, Serious games, App-based, Gamification

## Abstract

**Background:**

Virtual reality exposure therapy (VRET) has been shown to be as effective as traditional forms of in vivo exposure therapy for the treatment of specific phobias. However, as with in vivo exposure, VRET still involves relatively high costs and limited accessibility which makes it prohibitive for a large part of the population. Innovative methods using smartphone applications (apps) may improve accessibility and scalability of VRET. The aim of this study is to evaluate 0Phobia, a gamified self-guided VRET for acrophobia that is delivered through a smartphone app in combination with rudimentary cardboard virtual reality (VR) goggles.

**Methods/design:**

Participants (*N* = 180, aged 18–65 years) with acrophobia symptoms will be recruited from the Dutch general population and randomized to either 0Phobia (*n* = 90) or a waitlist control condition (*n* = 90). 0Phobia will be delivered over a period of 3 weeks and includes psychoeducation, VR exposure, cognitive techniques, monitoring of symptoms, and relapse prevention. The primary outcome measure will be the Acrophobia Questionnaire. Secondary outcome measures will include user-friendliness, symptoms of anxiety, depression, and mastery. Assessments will take place online at baseline, directly after the intervention (post test) and at follow-up (3 months).

**Discussion:**

This study capitalizes on novel technology and recent scientific advances to develop an affordable and scalable treatment modality.

**Trial registration:**

Netherlands Trial Register: NTR6442. Registered on 29 June 2017.

**Electronic supplementary material:**

The online version of this article (10.1186/s13063-018-2704-6) contains supplementary material, which is available to authorized users.

## Background

With lifetime prevalence estimates of between 8 and 15% [1, 2], specific phobias rank among the most prevalent mental disorders, along with depressive disorders and social phobia [3]. The lifetime prevalence of specific phobias in the Netherlands is nearly 8%. Around 530,000 people in the Netherlands currently suffer from one or more specific phobias and each year there are 75,000 new cases with a specific phobia diagnosis [3]. In the general EU population, 18.5 million people are estimated to suffer from a specific phobia [4]. Acrophobia is the most prevalent of all specific phobia subtypes [5, 6]. Due to high treatment costs, long waiting lists, and a general reluctance to seek treatment [7], access to evidence-based therapy is currently limited. If left untreated, specific phobias can become chronic and increase the risk of developing other mental disorders, such as anxiety and depression, especially for women [8]. Given the psychological burden that phobias carry, along with an increased risk of developing comorbid depressive and anxiety disorders, and the economic burden for society [9], there is an evident need for affordable and scalable self-help interventions.

Specific phobias have a lengthy history of clinical research and effective treatment exists [10]. In exposure therapy a person is gradually exposed to the object or situation of their fear. Meta-analyses have repeatedly demonstrated the effectiveness of exposure to reduce anxiety levels of specific phobias in different settings (e.g., [10, 11]). In fact, exposure therapy is among the most effective treatments existing in psychological health care [10].

### Virtual reality exposure therapy (VRET)

Over the past decades, a new type of treatment based on the principles of exposure therapy has emerged. This new form of exposure therapy relies on virtual reality (VR) rather than exposure “in vivo.” In VRET, artificially created computer-generated environments replace real-life settings. Individual studies as well as meta-analyses on treatment effectiveness for people suffering from anxiety disorders, and specific phobias in particular, have shown VRET to be as effective as traditional forms of exposure therapy in reducing anxiety (e.g., [12–17]). For example, in a recent meta-analysis of 14 studies [13], participants in the VRET condition for specific phobias improved significantly on behavioral assessments after VRET from pre to post test (aggregated uncontrolled effect size *g* = 1.23) as well as when compared with waitlist control subjects (*g* = 1.41). Additionally, there were no significant differences in terms of effectiveness between VRET and exposure in vivo at post test and follow-up (*g* = − 0.09 and 0.53, respectively), although results should be interpreted with caution as these studies were not adequately powered. Furthermore, in a meta-analysis by Opriş et al. [16], results demonstrated that VRET has better outcomes than waitlist controls, similar efficacy for VRET and cognitive behaviour therapy (CBT), and no difference in dropout rate between VRET and traditional exposure in vivo.

Aside from demonstrated effectiveness for anxiety disorders, and specific phobias in particular, VRET has a number of additional advantages over traditional “in vivo” treatment, such as the possibility to conduct it within the confines of the therapist’s office rather than having to go outside. Furthermore, VRET offers more flexibility in terms of sequencing, intensity of treatment, and graduality of exposure [18]. That is, people can practice more often and with a larger variety of scenarios compared to in vivo exposure, which may optimize exposure therapy [19]. Also, in VR, serious gaming elements can be integrated during exposure, which may reduce distress as compared with traditional exposure therapy [20]. Continuous confrontation with phobic stimuli may be facilitated by a gamified VR content that can be played repeatedly [20, 21].

Nonetheless, in spite of having several advantages over traditional in vivo therapy, VRET still involves relatively high costs and limited accessibility which make it prohibitive for a large part of the population. Existing VRETs tend to require heavy graphic processing capabilities not found in ordinary computers and mobile devices. Finally, and importantly, existing VRETs still require the intervention of a therapist.

### Mobile app interventions

Recent research has demonstrated the efficacy of mobile-app-based therapies for anxiety and depression [22, 23]. App-based mental health interventions based on CBT principles have shown to be effective in reducing mental health symptoms (e.g., [23] for a review) and guided Internet interventions have proven potential to be cost-effective ([24] for a review, [25]).

Advantages of app-based interventions compared to traditional therapy include high accessibility, real-time progress monitoring, portability, flexibility, and cost-effectiveness [26]. To the best of our knowledge, so far only one study has investigated the effectiveness of guided VRET using a mobile application and VR goggles. This intervention led to a significantly reduced fear of spiders [27], although older technology (red/blue anaglyph glasses and a computer monitor) was used. Another study, in which the effectiveness of guided VRET on a smartphone and VR goggles compared with traditional one-session exposure therapy for subjects with spider phobia, is currently being evaluated [21]. To our knowledge, no studies have yet explored the feasibility and efficacy of (1) self-guided VRET delivered through a smartphone app using (2) low-cost VR goggles for acrophobia. Instead of high-end head mounted displays (e.g., Samsung Gear VR), we use rudimentary 10-dollar cardboard VR goggles. The aim of this project is to test a self-guided, stand-alone treatment modality for acrophobia symptoms through exposure therapy by integrating VR technology with a smartphone app. The acrophobia intervention will be tested for user-friendliness as well as its effectiveness in reducing acrophobia symptoms for adults from the general Dutch population with acrophobia symptoms using a randomized controlled trial (RCT) design. We hypothesize that participants in the experimental condition will show a significant reduction in acrophobia symptoms at post test and follow-up compared to baseline and significantly less acrophobia symptoms than the waitlist control condition.

## Methods/design

### Study design

**Fig. 1:**
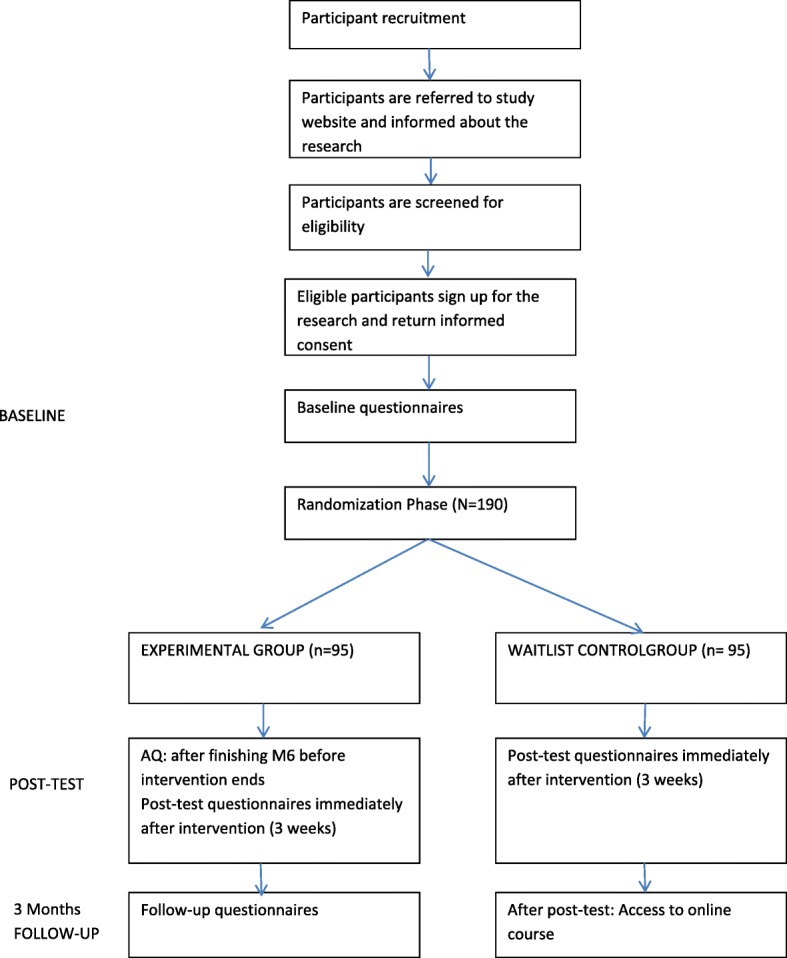
Study flowchart

A RCT will be carried out, in which the efficacy and user-friendliness of a self-guided, 3-week, mobile-app-based VR self-guided treatment “0Phobia” will be evaluated. In total, 180 participants from the Dutch general population will be randomized to two conditions: the experimental condition (0Phobia; *n* = 90) and a waitlist condition (*n* = 90). Measures will be taken at baseline and directly after the intervention (post test). After post-test assessment, the waitlist group will be granted access to the intervention. The experimental condition will receive a final follow-up questionnaire 3 months after baseline (follow-up). All measures will be completed online. Two reminders will be send (one by email, one by telephone) if necessary. This study has received ethical approval by the Medical Ethics Committee of the VU University Medical Center (registration number 2016–563) (Trial registration: NTR 6442). Figure 1 presents the flowchart of the study.

### Procedure

Advertisements with a call to participate in a VR mobile app study for acrophobia will be posted on several websites (e.g., proefpersonen.nl, Facebook) and in magazines (e.g., VU magazine). Media coverage in national television and radio broadcasts will provide additional publicity for the study. Potential participants are directed to the study website (http://www.0-phobia.nl) where information about the study is provided including eligibility criteria. Interested participants can leave their contact details at the website. The research team will send them a screening questionnaire to assess eligibility. Ineligible participants will be automatically directed to a screen explaining why they cannot participate in the study. The screening questionnaire assesses level of depression and suicidal thoughts, inclusion and exclusion criteria only. Participants excluded as a result of high levels of depression are advised to contact their general practitioner (GP). In case of high risk for suicide, participants are contacted by telephone by a member of the research team. The participant will be asked for permission to contact their GP.

Eligible participants receive an information letter and consent form with a return envelope. If necessary, they will be reminded by email to complete the informed consent (two reminders in total). After receiving the signed consent form, participants are invited to fill out an online screening battery. After completion, an independent researcher will assign participants to either the experimental condition or the waitlist condition based on the randomization list which is created by using Random Allocation Software. The participants will be send an email stating to which condition they are assigned. Participants assigned to the intervention condition will be sent the VR goggles by postal delivery – along with user instructions for the VR goggles, instructions to conduct the VR sessions (e.g., to start sitting and when anxiety decreases to stand up, to remove sharp objects in their vicinity to avoid injury, to practice each day at a set time), and instructions on how to download and use the 0Phobia app.

The app is accessed with a unique individual code on the participant’s own smartphone. Once the app is installed, participants can begin with 0Phobia intervention which they can follow for 3 weeks at their own pace. During this 3-week period participants receive weekly motivational emails with information reminding them to start or continue with 0Phobia to increase intervention adherence. These emails will be sent unconditional of app activity.

If participants have questions or experience severe distress, they are encouraged to contact the research team.

After module 6, participants are encouraged to seek out heights in real life. Therefore, participants who finish 0Phobia who have finished module 6 earlier than the 3 weeks will receive an email with a link to the Acrophobia Questionnaire (AQ [28]) in order to obtain estimates of the treatment on levels of acrophobia through VR exposure.

Directly following the conclusion of 0Phobia, participants are sent a link to the online post test. After completing the post test, the waitlist condition will be granted access to the 0Phobia therapy. After 3 months, participants in the experimental condition are asked to fill in the follow-up questionnaire.

### Inclusion and exclusion criteria

A sample of 180 individuals (aged 18–65 years) with symptoms of acrophobia will be recruited among the Dutch population. Inclusion criteria are a sufficient level of AQ-Anxiety (AQ score of 45.45, which is one standard deviation below the mean of a previous acrophobic sample; [28, 29]) having access to an Android smartphone (Android v.5.1 Lollipop or higher, 4.7–5.5-in. screen and gyroscope) with Internet, and having provided informed consent. Participants outside the 18–65 years age range, participants with insufficient knowledge of the Dutch language, those under treatment for a specific phobia or taking psychotropic medication (unless on stable dosage for the previous 3 months and no changes planned during the study period), and participants with symptoms of severe depression (Patient Health Questionnaire (PHQ-9); total score > 19) or suicidality (Web Screening Questionnaire; WSQ, score 3; [30]), are excluded from participation. Suicidal ideation will be measured using one item about suicidal ideation from the Screening Questionnaire [31] that has been translated for the Dutch population in the WSQ [30]: “has the idea of harming yourself, or taking your own life, recently come into your mind?” Answer options are: (1) “Definitely not”; (2) “I seriously considered it but I stopped myself”; (3) “I would do it given the opportunity.” Participants with a score of 3 will be excluded from participation and will be contacted by a member of the research team.

### Sample size

The primary outcome measure, the AQ, has been used for the power calculations. In previous RCTs using the AQ as an outcome measure, effect sizes of 0.79–1.42 [14, 15] were demonstrated. However, as the present intervention will use low-end cardboard VR goggles instead of expensive head-mounted displays, the current study uses a more conservative estimate for the post-treatment effect on the AQ. In order to detect a difference between the experimental and the waitlist control condition with a standardized effect size (Cohen’s d) of 0.50 (two-sided), an alpha of 0.05 and statistical power (1 − *β*) of 0.80, we need 64 participants in each condition (128 participants in total). With an anticipated dropout of 40%, this requires a total target sample size of 180 participants.

### Randomization, blinding, and treatment allocation

A randomization list will be created with Random Allocation Software using block randomization of 6, 8, 10, and 12 blocks at an allocation ratio 1:1. Participants will be randomized into two groups: experimental or waitlist condition. The randomization list is kept by an independent researcher. This person reveals the next randomization outcome after every inclusion and thus ensures that the researchers are blind for treatment allocation. Due to the nature of the study, it is not possible to blind the participants for the randomization outcome.

### Intervention: 0Phobia

**Fig. 2:**
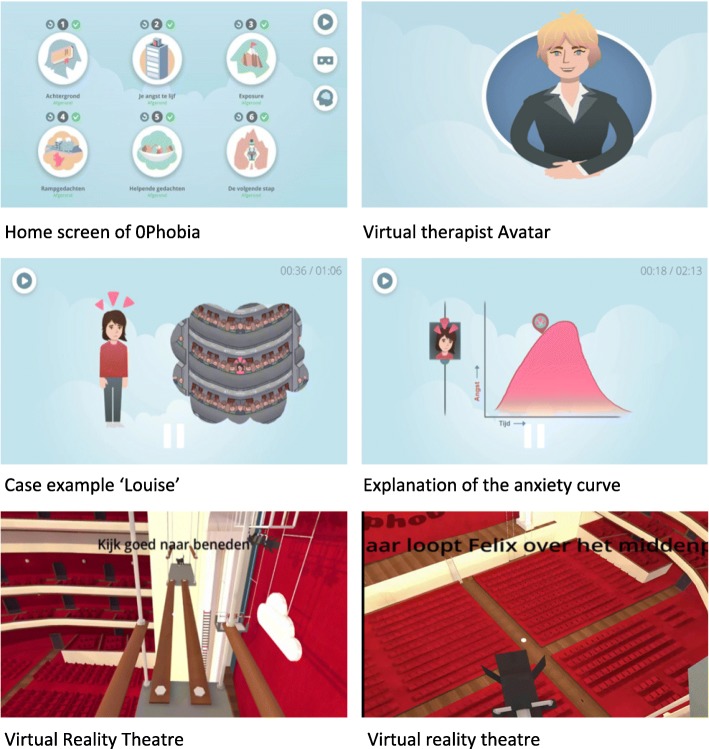
Screenshots of 0Phobia

0Phobia consists of six animated cognitive behavioral therapy (CBT)-based modules. The home screen (Fig. 2) features the six modules, each of which is consecutively unlocked, a “watch again” menu in which each of the instructions can be watched again, a menu providing access to the VR environment, and a “My 0Phobia” menu where participants can view the goals they have set for themselves for the intervention, their dysfunctional and functional thoughts, and their anxiety hierarchy for practicing in the real world.

**Fig. 3:**
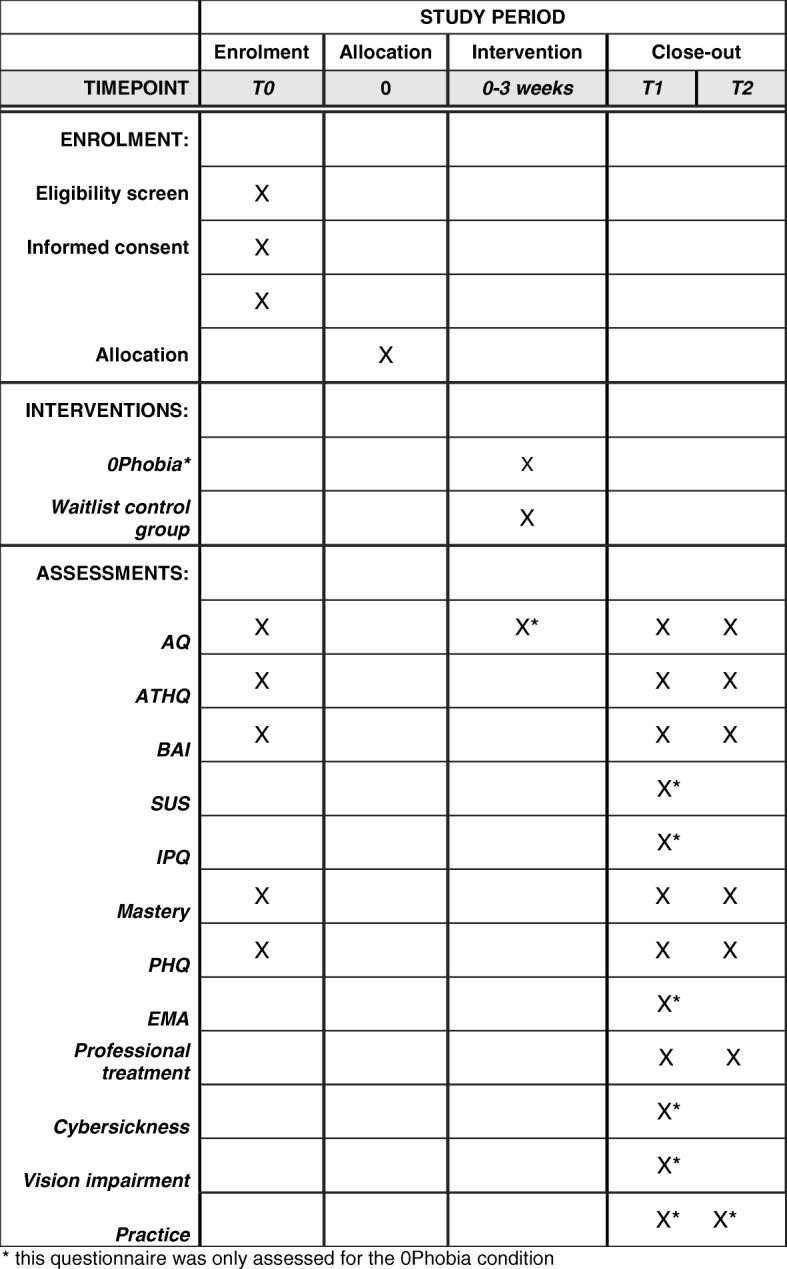
Standard Protocol Items: Recommendations for Interventional Trials (SPIRIT) schedule for enrollment, interventions and assessments

Participants will follow the modules according to their own tempo and timing. Each module modules takes between 5 and 20 min to complete (see Table 1). The CBT content in the modules is provided by a virtual therapist using 2D animations and a voice-over (Fig. 2). The virtual therapist, “Tara,” guides the participant through the intervention and provides case examples in the form of a returning character, “Louise,” a theater manager who has overcome her own acrophobia (Fig. 2). Over the course of the modules Tara describes to Louise how her phobia emerged and developed, what the consequences were for her and how she resolved it (i.e., through exposure therapy and repeated practice, see Fig. 2). Louise also motivates the user to continue with 0Phobia.

**Table 1:** Overview of 0Phobia modules

Modules	Contents
1. Psychoeducation	Explanation of the nature of a specific phobia, its emergences and potential consequences
2. Goals and treatment principles	Participants define their goals for the treatment and treatment principles underlying exposure are explained
3. Exposure therapy	Virtual reality (VR) content will be explained and participants will practice in a virtual environment
4. Cognitive therapy	According to the established principles of cognitive behavioral therapy (CBT), users identify and evaluate their automatic catastrophic thoughts regarding heights (e.g., “I am bound to fall” or “I will jump”). After that, subjects can practice with non-interactive 360° VR videos
5. Cognitive therapy	In this module, users will develop helping thoughts countering the automatic catastrophic thoughts identified in module 4
6. The next steps..	This module contains information on how the user can continue their practice and further reduce their fear and prevent relapse. This includes developing an individualized fear hierarchy. Explicit attention is devoted to motivation and encouragement

Aside from the 2D animated environment, the intervention consists of a gamified immersive VR environment. From module 3 onwards, participants start to practice with exposure in the virtual environment, parallel to completing the remaining modules. The virtual environment is a gamified theater setting and within the story of the game, the participant is replacing Louise as the theater manager (see Fig. 2). To prepare the theater for the night’s performance, participants need to complete a number of assignments in the theater. Each of these assignments involve different levels of exposure to heights (from standing on a small ladder to standing on a ledge high above the stage). By looking at assets located on the theater floor that need to be collected, participants are encouraged to look down and face their fears.

Aside from the virtual theater, 0Phobia also contains four 360° videos that are also watched with the VR goggles in which participants stand on the top of a high building, cross a high suspension bridge, sit on a rooftop with their legs dangling over the edge, or stand on a high crane. In conjunction, the various virtual environment and 360° videos cover the entire exposure spectrum, from very-low- to very-high-intensity exposure. Based on the fear levels and performance the user receives feedback. When users rate their fear levels using a stare function in VR below a score of 3 on a 10-point scale, an automated feedback pops up mentioning that they can continue to the next level. When users rate their anxiety level between 4 and 7, they are advised to practice the same level again. When rated an anxiety level between 8 to 10, they need to practice the same level again before commencing to the next level. Subjects can return to practicing in VR as often as they like and gradually expose themselves to different heights and fear intensities. Although the importance of exposure in VR is stressed out in the 2D animations and participants are encouraged to practice in VR, participants can skip VR exposure and proceed to the next module, because for some people, fear levels may still be too high. After evaluating their automatic anxiety evoking thoughts, they can practice in VR exposure. Only during the exposure sessions will participants use the cardboard goggles.

Participants will have access to the mobile app after post test and follow-up. Usage data during this period will be collected. 0Phobia has been extensively user-tested throughout development process and modified according to user-feedback.

### Assessments

#### Primary outcome measure

##### Acrophobia questionnaire

The main outcome measures will be the 20-item anxiety subscale and the 20-item avoidance subscale of the Acrophobia Questionnaire (AQ; [28]). The AQ is a widely-used instrument with good psychometric properties [28]. The anxiety subscale is measured using a 7-point Likert scale (0 = not anxious to 6 = extremely anxious), total score range 0–120. The avoidance subscale uses a 3-point Likert scale (“I would not avoid it” to “I would not do it under any circumstances”). Both subscales will be taken at baseline, directly after finishing the modules, at post test, and at follow-up. See Fig. 3. 

#### Secondary outcome measures

##### Attitudes Towards Heights Questionnaire

The Attitudes Towards Heights Questionnaire (ATHQ; [33]), with minor modifications to the wording reported in [34] is a six-item measure in which individuals read pairs of dichotomous adjectives describing ways people may feel about heights (e.g., “Good/Bad,” “Safe/Dangerous”), and rate how they feel about elevated places on a scale of 0 (which corresponds with the first adjective) to 10 (which corresponds with the second adjective). The ATHQ has been used in several acrophobia treatment studies and is sensitive to treatment effects [34, 35]. The reliability is good [36]. This questionnaire will be completed at baseline, post test, and follow-up.

##### Beck Anxiety Inventory

The Beck Anxiety Inventory (BAI; [37]) is a 21-item self-report questionnaire assessing symptoms of anxiety. Participants record how much they have been bothered by each symptom during the past week, including the day the questionnaire is administered (4-point Likert scale ranging from 0 = not at all to 3 = severely: “I could barely stand it”). The total score ranges from 0 to 63. Internal consistency is high (0.90–0.94) and convergent validity is good [38]. This questionnaire will be completed at baseline, post test, and follow-up.

##### System Usability Scale

The System Usability Scale (SUS; [39]) will be used to measure the user-friendliness of the app. The SUS is composed of 10 statements that are scored on a 5-point Likert scale ranging from 1 = strongly agree to 5 = strongly disagree. Scores are converted to a new number, added up and then multiplied by 2.5 to convert the original scores to 0–100, with higher scores indicating better usability (for details, please see Bangor et al., 2008). This means that digital products that are at least passable have SUS scores above 68, with better products scoring in the high 70s to upper 80s. Truly superior products score above 90. Products with scores less than 70 should be considered candidates for increased scrutiny and continued improvement and should be judged to be marginal at best. Reliability of the scale is good [39]. This questionnaire will be completed at post test.

##### Igroup Presence Questionnaire

The Igroup Presence Questionnaire (IPQ; [40]) is a 14-item questionnaire which assess realism and “presence,” i.e., the subjective feeling of being immersed in the virtual environment. Each of the items has five response categories from 1 = fully disagree to 5 = fully agree. Internal consistency is adequate. This questionnaire along with some open questions about user experience of the VR environment will be completed at post test.

##### Mastery

The Pearlin Mastery Scale [41] is a seven-item scale to measure self-experienced control over a situation. Each of the seven items has five response categories ranging from 1 = totally disagree to 5 = totally agree. The questionnaire has good psychometric properties [41]. This questionnaire will be completed at baseline, post test, and follow-up.

##### Patient Health Questionnaire

The nine-item mood module of the Patient Health Questionnaire (PHQ-9; [42]) is used to screen subjects for depressive disorders. The nine items are each scored on a 4-point Likert scale ranging from 0 to 3 (total score range 0–27). Sensitivity and specificity coefficients are good [43]. This questionnaire will be completed at baseline, post test, and follow-up.

##### Ecological momentary assessment

Assessment of current anxiety level directly after exposure when using the 0Phobia app (one question: ‘How high was your anxiety at highest?’).

#### Additional measures

##### Professional treatment

Two items will be included in the screening, the post-test, and the follow-up measure assessing whether participants have attended professional treatment or received medication for their specific phobia right before (screening), during (post test), or after (follow-up) the 0Phobia study.

##### Cybersickness

Participants will rate the extent to which they experienced cybersickness while practicing in VR using three items adapted from the Simulator Sickness Questionnaire, (SSQ; [44, 45]), e.g., “How often did you experience any of the abovementioned symptoms while practicing in virtual reality” using a 3-point scale ranging from 0 (never) to 3 (always). Symptoms include eye strain, headache, pallor, sweating, dryness of mouth, fullness of stomach, disorientation, vertigo, ataxia, nausea, and vomiting [44]. This questionnaire will be completed at post test.

##### Vision impairment

Participants will be asked to report any potential vision impairments they have (e.g., wearing glasses, lazy eye, cataract, other). This questionnaire will be completed at post test.

##### Practice in real life

Two items will be used to determine the extent to which participants also practiced with height situations in real life. This questionnaire will be completed at post test and follow-up.

All assessments are programmed with Survalyzer software [46] (Fig. 3).

### Analyses

#### Statistical analyses

The primary outcome measure (AQ; [28]) and secondary study parameters (ATHQ, PHQ, IPQ, SUS, BAI, and Pearlin Mastery Scale) will be treated as continuous outcomes. Continuous variables will be presented as mean, standard deviation, and minimum and maximum number of observations. Categorical variables will be presented in terms of frequencies and percentages. Descriptive statistics of demographics and clinical outcomes will be compared between the experimental and the waitlist condition.

To assess whether attrition is non-random, we will construct a balancing table for background characteristics, pre-scores and other covariates in which participants with and without missing outcome observations are compared. Potential implications of non-random sample attrition on the observed treatment estimates will then be assessed by estimating treatment effect bounds for samples with non-random sample selection/attrition as proposed by Lee [47]. Using this approach we are able to infer whether observed differences between the intervention and control groups can be explained by extreme, non-random sample selection. Depending on these results, missing outcome observations for participants will be imputed based on pre-scores and a set of background characteristics. The quantitative analysis of the primary and secondary endpoints will be performed on an intention-to-treat (ITT) basis following the per-protocol analysis. The primary analysis will be based on ITT. Potential dependency between outcome measures is addressed using multivariate analyses. Potential heterogeneity will be addressed by estimating treatment effects for subgroups (e.g., based on pre-scores). In order to understand the mechanisms by which observed differences are generated, a rich set of analyses is performed based on variation in (1) experiences when using the app (e.g., perceived user friendliness, ecological momentary assessment, cybersickness, VR vs 360°) and (2) intensity of using the app. Per-protocol analysis will be based on two groups: (1) those who returned the post-test and follow-up questionnaires (completers) and (2) those who completed 50% or more of the treatment modules and returned the questionnaires (adherent completers). Comparisons will be made between and within the groups before and after measurements.

Intention-to-treat and per-protocol analysis will be used on continuous scales using regression estimation standardized effect sizes (Cohen’s *d*) and confidence intervals will be calculated. Usage data through the mobile app (anxiety level during VR exposure, time spent in VR, number of VR sessions completed) will be collected during the study to model missing data. SPSS version 21 will be used for the analyses. A *p* value < 0.05 will be considered to indicate statistical significance. All design, implementation, and reporting will be carried out in accordance with Consolidated Standards of Reporting Trials (CONSORT) and Standard Protocol Items: Recommendations for Interventional Trials (SPIRIT) guidelines (Additional file 1) [48–50].

### Data monitoring and management

All data will be collected at the Section Clinical Psychology of VU University of Amsterdam by a staff member and handled confidentially. The data (baseline, AQ after module 6, post test, and follow-up data) will be captured electronically in a secured online survey platform [46]. Through the 0Phobia app the following data is collected: anxiety level during VR exposure, time spent in VR, number of VR sessions completed, and information from three exercises (setting goals for the intervention, evaluating anxiety provoking thoughts and creating a personal fear hierarchy). The data will be coded which serves as a trial identifier consisting of four numbers and captured electronically in a secured online database, which is hosted on a server of our university. No personal identification data will be collected through the app. The data will be uploaded into the IBM SPSS database. Data from the different measurements will be kept in separate databases and merged into a master database only after data collection is completed and each individual database is locked. Data will be coded and the key connecting names to numbers will be kept in a separate, secure location in the principal investigator’s office. The data will be anonymized but linked with the trial identifier consisting of four numbers. Coded data will be electronically stored at the VU Amsterdam, separate from identifying information. Access will be password protected. Only the principal investigators and trial researchers will have access to the final dataset. All collected data will be used only for the purposes of this research. The project group will analyze the data, and both positive and negative trial results will be disclosed. The publication policy is in agreement with the publication statement of the CCMO (see: http://www.ccmo.nl). A data monitoring committee is not required by the Ethics Committee because of the low impact on the safety of the participants. No interim analysis is anticipated as this is not required for a trial of this type.

### Harms

Previous studies in similar samples have shown that studies with VRET can be carried out safely, without a significant risk for unwanted effects (e.g., [18, 27, 32, 51]).The VR exposure environment uses gradual exposure which means that subjects start with relatively easy levels of height situations which induce a small amount of fear. When this situation becomes less fearful, they move on to the next level. In this way, fear levels are manageable. It is, nevertheless, possible that participants may get distressed, cybersick, or feel that they will lose their balance and fall during the intervention. In these cases, participants are instructed to remove their VR goggles. By removing them, feelings of distress, cybersickness or imbalance are immediately reduced. Furthermore, participants may experience distress while completing questionnaires when asked about mental health symptoms. However, administering these instruments is crucial to draw conclusions about the feasibility and efficacy of the intervention. In case of an undesirable emotional reaction both during the intervention as well as during the follow-up assessments, the research assistant and at least one experienced clinician (the principal investigator) can be contacted by the participant and will be available to provide support if necessary or desirable. Participants can always discontinue the intervention without providing any reasons.

### Protocol amendments and publication

If necessary, protocol amendments will be submitted to the Ethics Committee and specified in the trial registry. There are no restrictions on reporting findings of this trial. Findings will be published in peer-reviewed journals.

## Discussion

The aim of this study is to evaluate effectiveness and user-friendliness of a self-guided VRET for acrophobia delivered through a smartphone app using cardboard VR goggles. Due to high treatment costs, long waiting lists, lack of health insurance coverage in the Netherlands, and a general reluctance to seek treatment, access to evidence-based therapy is currently limited. Given the psychological burden that phobias carry, the increased risk of developing comorbid depressive and anxiety disorders [8], and the heavy economic burden for society [9], there is a strong need for affordable and scalable self-guided interventions. Recent research into mobile apps as a method for treating psychiatric disorders are promising (e.g., [23, 52, 53]) and research into VRET as a treatment technique for specific phobias has shown positive results (see [13] for a review). The combination of a mobile app with affordable VR goggles is an innovative and promising scalable solution to deliver clinically validated mental health care for people suffering from specific phobias such as acrophobia. Advantages of an app-based VRET are lower costs, no waiting lists, better accessibility and participant retention, real-time progress monitoring, portability, and flexibility. Using a gamified exposure application, subjects may be more engaged and less distressed compared to traditional exposure therapy. Game elements may reduce distress as compared with traditional exposure therapy [20]. With the possibility of playing the gamified VR contact repeatedly, continues confrontation of phobic stimuli may be facilitated [21].

In sum, the current study will assess the efficacy and user-friendliness of 0Phobia, a mobile-app-based gamified VR treatment for acrophobia, in a RCT. This study will add to the development of innovative and scalable delivery methods of evidence-based treatments.

### Trial status

Recruitment started March 2017 and is ongoing at the time of initial manuscript submission (June 2017).

## Additional file

Additional file 1: Standard Protocol Items: Recommendations for Interventional Trials (SPIRIT) 2013 Checklist: recommended items to address in a clinical trial protocol and related documents. (DOC 121 kb)

## Abbreviations

ANOVA: Analysis of variance
AQ: Acrophobia Questionnaire
ATHQ: Attitudes Towards Heights Questionnaire
BAI: Beck Anxiety Inventory
CBT: Cognitive behaviour therapy
GLM: Generalized linear models
IPQ: Igroup Presence Questionnaire
PHQ: Patient Health Questionnaire
RCT: Randomized controlled trial
SPSS: Statistical Package of Social Science
SUS: System Usability Scale
VR: Virtual reality
VRET: Virtual reality exposure therapy
VU: VU University Amsterdam
WSQ: Web Screening Questionnaire
